# Alcohol use, intentions to reduce consumption, and perceived motivations and barriers among cancer survivors

**DOI:** 10.3389/fpubh.2026.1832117

**Published:** 2026-06-22

**Authors:** César Alas-Pineda, Christine M. Gunn, Judy R. Rees, Jennifer A. Emond

**Affiliations:** 1 Quantitative Biomedical Sciences Doctoral Program, Geisel School of Medicine, Dartmouth College, Hanover, NH, United States; 2 The Dartmouth Institute for Health Policy and Clinical Practice, Geisel School of Medicine, Dartmouth College, Lebanon, NH, United States; 3 Dartmouth Cancer Center, Geisel School of Medicine, Dartmouth College, Hanover, NH, United States; 4 Department of Epidemiology, Geisel School of Medicine at Dartmouth, Lebanon, NH, United States; 5 Department of Biomedical Data Science, Geisel School of Medicine, Dartmouth College, Hanover, NH, United States

**Keywords:** alcohol drinking, awareness, cancer survivors, risk reduction behavior, survivorship care

## Abstract

**Purpose:**

To characterize alcohol use patterns, awareness of alcohol's oncologic risks, and efforts and intentions to reduce consumption among cancer survivors in New Hampshire and Vermont.

**Methods:**

Cross-sectional survey of 471 cancer survivors aged ≥21 years enrolled in two state-wide, online panels. Participants reported their current or former alcohol use, completed the Alcohol Use Disorders Identification (AUDIT), and replied to a series of items regarding any efforts to reduce consumption and related motivations and barriers.

**Results:**

Among 471 survivors, 34.2% of which had an alcohol-related cancer, 65.8% were current alcohol users and 29.1% former alcohol users. Awareness that alcohol increases cancer risk was reported by 49.7% of current and 48.2% of former alcohol users. Among current alcohol users, 26.5% would like to reduce consumption within the next 6 months, with this subset consuming a median of seven drinks/week and 23.2% having an AUDIT score ≥8. Negative impacts on cardiovascular health and memory were top health concerns related to alcohol use, while using alcohol to socialize and for stress relief were key obstacles to reducing consumption.

**Conclusion:**

A substantial proportion of cancer survivors consume alcohol, with limited awareness of its oncologic risks. Even among this sample of largely moderate alcohol users, a considerable portion wished to reduce their alcohol use. Educational and behavioral interventions targeting cancer survivors are needed to raise awareness about alcohol risks and address barriers to reducing alcohol consumption. The 2025 U.S. Surgeon General's Advisory identifying alcohol as the third leading preventable cause of cancer lends urgency to these efforts, and focusing on that role may improve overall survivorship.

## Introduction

Alcohol consumption is a leading modifiable behavioral risk factor associated with disease, disability, and mortality worldwide (1). It is also a well-established Group one carcinogen, implicated in cancers of the oral cavity, pharynx, larynx, esophagus, liver, colorectum, and breast (2–5). Globally, alcohol consumption is estimated to account for 4.1% of incident cancer cases (6) and between 3.5 and 5% of cancer-related deaths annually (3), underscoring its substantial impact on cancer epidemiology. In addition to its role in cancer development, alcohol use may adversely affect cancer prognosis, as alcohol consumption has been associated with poorer treatment outcomes, including reduced efficacy of therapeutic interventions and higher complication rates during treatment (7). Evidence also indicates that continued alcohol consumption increases the likelihood of cancer recurrence among breast, head and neck, and colorectal cancer survivors, as well as the development of new primary tumors and both cancer-specific and all-cause mortality (5, 8–11). Alcohol use also increases symptoms of depression and anxiety (12, 13), and cancer survivors may be highly vulnerable to those effects given their risk profile for those disorders. Indeed, alcohol is often used as a coping mechanism during survivorship (14), creating a self-perpetuating cycle of dependence in which emotional distress prompts alcohol consumption. This pattern undermines engagement with evidence-based psychosocial and lifestyle interventions and ultimately impedes efforts to mitigate alcohol's carcinogenic risk (15–17). In 2025, the U.S. Surgeon General issued an Advisory identifying alcohol as the third leading preventable cause of cancer in the United States, after tobacco and obesity, contributing to approximately 100,000 cancer cases and 20,000 cancer deaths annually (18).

Despite the risks associated with alcohol use, many cancer survivors continue to consume alcohol after their diagnosis. Recent studies have shown that approximately two-thirds of cancer survivors report current alcohol use, and about half of patients do not change their drinking patterns after a cancer diagnosis, with a significant proportion engaging in risky drinking behaviors, such as binge drinking or exceeding moderate consumption limits (11, 17, 19, 20). Furthermore, 38.3% of 15,199 cancer survivors in the All of Us Research Program met diagnostic criteria for hazardous drinking, raising concerns about their long-term health outcomes (19). One critical challenge in addressing alcohol use among cancer survivors is the lack of awareness about alcohol's role as a carcinogen. Studies indicate that only 37% of U.S. adults correctly recognize alcohol as a cancer risk factor, a gap in awareness that extends to cancer survivors themselves (15, 21, 22). Confusion about alcohol as a cancer risk factor is understandable: current guidelines and recommendations for alcohol use in relation to cancer remain inconsistent (23–25). While the World Health Organization has concluded that any alcohol consumption increases the risk of at least seven cancers (26), the 2025–2030 U.S. Dietary Guidelines removed specific daily alcohol limits and omitted cancer risk in alcohol guidance entirely, drawing criticism from multiple medical organizations (27).

Reducing alcohol intake after diagnosis may improve cancer outcomes among survivors (28). However, evidence for interventions specifically designed to reduce alcohol use in this population remain limited (29). Clinicians should discuss alcohol-related cancer risk, screen patients using validated instruments, and provide brief interventions for those exceeding recommended limits (28). Cancer survivors are an important population for these efforts because they may be at increased risk of recurrence and second primary tumors, have frequent contact with the health care system, and may be particularly receptive to behavior change after diagnosis (17); longitudinal data in breast cancer survivors further suggest that alcohol intake often declines during the first year of survivorship (30). Therefore, the goal of this study was to characterize alcohol use patterns, awareness of alcohol's role as a carcinogen, and barriers to reducing alcohol consumption among a sample of adult cancer survivors recruited statewide in New Hampshire and Vermont.

## Materials and methods

The overarching goal of the study was to evaluate alcohol consumption patterns and awareness of alcohol as a risk factor among adult cancer survivors residing in our Cancer Center's primary catchment area of New Hampshire (NH) and Vermont (VT). Data were collected through an online survey administered in September 2024. The study population consisted of adult cancer survivors identified through two established state panels: the Granite State Panel (NH) and the Green Mountain State Panel (VT), both managed by the University of New Hampshire (UNH) Survey Center. Both panels include approximately 10,000 residents. Residents opted into the panel by completing a brief UNH Survey Center questionnaire—either an online health-history survey or a short text-based survey after random cellphone sampling—and providing an email address for future studies (31). For this current study, an email invitation with a brief screening item was sent to all panelists; 1,126 panelists self-identified as having been diagnosed with cancer in adulthood and were eligible to complete the full survey, of whom 539 completed it (cooperation rate: 47.9%). The email invitation described the study as a survey about health behaviors and experiences among adults diagnosed with cancer; neither the invitation nor the survey content presented alcohol as a cancer risk factor before the relevant awareness item was administered. Eligibility criteria included being at least 21 years of age, residing in NH or VT for at least 6 months of the year, and having been diagnosed with cancer in adulthood (age ≥18 years). Individuals younger than 21 years were excluded to avoid potential biases in self-reporting of underage alcohol consumption. Of the 539 respondents, 68 were excluded because their sole cancer diagnosis was keratinocyte carcinoma (formerly non-melanoma skin cancer). This decision aligns with studies of invasive cancer epidemiology that have excluded individuals with KC due to its distinct etiology, generally favorable prognosis, and its exclusion from cancer reporting mandates (32, 33). As such, the final analytic sample consisted of 471 participants. All participants who completed the survey were entered into a raffle to win one of three $100 gift cards provided by the Survey Center. All study procedures were approved by the Committee for the Protection of Human Subjects at Dartmouth College and the University of New Hampshire.

Participants self-reported their primary cancer type from a dropdown list of 13 options; participants were asked to write in any additional type not on the list. Primary cancer types were classified as alcohol-related based on the International Agency for Research on Cancer (IARC) classification (34): Cancers of the oral cavity, pharynx, larynx, esophagus, liver, colon, and breast. Participants reported their age when diagnosed with their primary cancer to compute time since diagnosis. Participants reported if they were currently undergoing treatment for their first cancer or if they experienced a recurrence, new cancer, or metastasis. Participants completed the Functional Assessment of Cancer Therapy–General–seven Item Version (FACT-G7), a validated tool to assess cancer-related physical, functional, and emotional wellbeing (35). A final score is computed as the sum across the seven items. FACT-G7 scores range from 0 to 28, with higher scores indicating better quality of life.

Participants reported if they currently drink alcohol (yes/no); those who answered no were asked if they drank alcohol in the past. Never alcohol users were defined as those who do not drink currently and did not drink in the past. Current consumers reported how many drinks per week they consume in a typical week; past consumers reported how many drinks per week they consumed in a typical week when they used to drink alcohol. Exceeding moderate drinking was defined as more than 14 drinks per week for men or more than seven drinks per week for women, which aligns with the 2020–2025 USDA dietary guidelines of no more than two drinks per day for men or one drink per day for women. Current consumers also completed the Alcohol Use Disorders Identification Test (AUDIT), a clinically validated screener for alcohol use disorders (36). A final score is computed, ranging from 0 to 40. AUDIT scores ≥ 8 are suggestive of hazardous drinking, and scores ≥ 15 suggest alcohol dependence (36). Engaging in binge drinking was defined as ever vs. never using the AUDIT item, *How often do you have six or more drinks on one occasion?* Responses of less than monthly, monthly, weekly, daily, or almost daily were defined as ever.

Participants were asked: *Before today, did you know that drinking alcohol could increase the risk of certain cancers*? Response options included yes, maybe, and no. Those who responded *yes* were defined as those who knew alcohol was a cancer risk factor. Separately, participants reported how a series of eight lifestyle behaviors affected cancer risk *(increased, decreased, no effect, don't know)*. Three of those items related to alcohol (*drinking beer, drinking wine*, or *drinking liquor/spirits*).

Current and former consumers reported if a health care provider had ever discussed ways to reduce or quit drinking alcohol, either in general or as part of cancer care *(no, yes, don't remember)*, and if they had ever reduced or stopped drinking alcohol because alcohol is a risk factor for certain cancers *(yes, a little bit, no)*. Participants further reported if they had ever made any efforts in the past to reduce their alcohol use *(yes, no)*. Current consumers reported if they would like to reduce their alcohol consumption in the next 6 months (*no, not sure, maybe yes, definitely yes*). Those who replied *maybe yes* or *definitely yes* were defined as having intentions to reduce their alcohol use, as informed by the contemplation phase of the transtheoretical model of behavior change (37).

A novel set of questions was also included to address motivations to reduce alcohol use based on physical, psychological, and social outcomes as defined by the US Centers for Disease Control and Prevention (38). Items covered the following domains: high blood pressure/cardiovascular disease and other medical problems; weakening of the immune system; learning and memory problems; mental health problems including depression and anxiety; social problems including family, job-related problems, and unemployment; and alcohol use disorders or dependence. These items were administered near the end of the survey and prefaced with *Too much alcohol use can also lead to other long-term health effects that are listed below. Please rate how important each item is to you as a reason to reduce or stop drinking alcohol*. Responses for each item were *not at all important, slightly important, moderately important, very important*, or *extremely important* and were dichotomized as important (*very important* or extremely *important*) vs. other. Current consumers also completed 12 items regarding barriers to reducing alcohol use; 11 items were taken from the Barriers to Alcohol Reduction (BAR) scale (39). The BAR is a 12-item scale developed and validated among a college population with a past-month history of binge drinking (39). One item in the BAR was not included for this survey (“*I feel like a treatment program to help me drink less would not have a consistent therapist for me to see”*). All other items were included, with any reference to “treatment programs” replaced with “programs.” This current study also added a new item, *I don't feel I need to drink less*, it was added to capture perceived need for behavior change. Responses for all items were *strongly disagree, somewhat disagree, neither agree nor disagree, somewhat agree, or strongly agree* and collapsed to a dichotomous outcome of agree (*strongly agree* or *somewhat agree*) vs. other.

Sociodemographic variables collected include age, sex, ethnicity, race, education level, marital status, household income, and residential zip code. Zip codes were used to classify the rural-urban status of communities using Rural-Urban Commuting Area (RUCA) codes (40). RUCA codes were collapsed into metropolitan (1–3), micropolitan (4–6), or rural (7–10) (40). Participants reported the number of days per week they engaged in moderate-to-vigorous physical activity and minutes of engagement on those days; values were multiplied to compute minutes per week of Moderate to Vigorous Physical Activity (MVPA) (41). Finally, participants who smoked at least 100 combustible cigarettes in their lifetime and currently smoked were considered current smokers; those who had smoked at least 100 cigarettes, but did not currently smoke, were classified as former smokers. Participants who had smoked fewer than 100 cigarettes in their lifetime or who had never smoked were classified as never smoker (19).

Descriptive statistics, including frequencies, percentages, means, medians, and interquartile ranges (IQR), were used to summarize participant characteristics. Bivariate associations between participant sociodemographic or cancer characteristics and current alcohol consumption were completed with chi-square tests, two-sample *t* tests, or Wilcoxon rank sum tests, as appropriate. Similarly, bivariate analyses tested the associations between awareness of alcohol as a cancer risk factor and past efforts to reduce drinking between former and current alcohol consumers, to test if those factors correlated with alcohol cessation. Bivariate analyses were used to further test the association between alcohol use metrics, barriers to reducing use, and motivations for reducing use, between current consumers with or without intentions to reduce drinking, to quantify what differentiates current alcohol users ready to reduce their alcohol intake. Additionally, analyses were repeated stratified by whether the primary cancer was alcohol-related (per IARC classification) to asses repeated differences in awareness, provider counseling, alcohol-use patterns (current use; AUDIT total and AUDIT ≥8), intention to reduce within 6 months, and quality of life (FACT-G7); categorical outcomes were compared using chi-square tests and continuous outcomes using two-sample *t* tests or Wilcoxon rank-sum tests. These stratified analyses were considered exploratory. The threshold of statistical significance was *p* < 0.05. All analyses were completed with the R Language and Environment for Statistical Computing, version 4.3.3.

## Results

### Sociodemographic and clinical characteristics

A total of 471 cancer survivors participated in the survey. Participants had a mean age of 69.4 years (SD = 9.9), with a range of 23 to 94 years. More than half of the sample identified as female (52.4%), nearly all participants self-identified as White (99.8%), and most participants reported living comfortably on their present income (60.3%). Educational attainment was high, with nearly half (47.6%) holding postgraduate degrees. The majority were married or in a domestic partnership (73.4%), and approximately half resided in metropolitan areas according to RUCA classification (48.4%). Regarding behavioral risk factors, 2.5% of participants were current smokers, while 38.6% were former smokers. Physical activity levels, measured by median minutes per week of MVPA, were 150 min (IQR: 80–280; Table 1).

**Table 1:** Sociodemographic and cancer history characteristics of cancer survivors in New Hampshire and Vermont (*n* = 471).

Participant characteristics	Overall *n* (%)
Age in years, mean ± SD	69.4 (9.9)
Female	247 (52.4)
Hispanic ethnicity	14 (3.0)
Race	
White	470 (99.8)
Black or African American	1 (0.2)
American Indian or Alaska Native	6 (1.3)
Marital status	
Married/domestic partnership	346 (73.4)
Divorced, separated, or widowed	94 (20.0)
Single	27 (5.8)
Prefer not to say	4 (0.8)
Education	
High school or less	20 (4.2)
Technical or post-secondary education	76 (16.1)
College graduate	151 (32.1)
Postgraduate education	224 (47.6)
Annual household income	
< $34,999	27 (5.7)
$35,000–$49,999	23 (4.9)
$50,000–$74,999	69 (14.7)
$75,000–$99,999	77 (16.4)
$100,000–$199,999	141 (29.9)
≥$200,000	51 (10.8)
Prefer not to say	83 (17.6)
Perception of income	
Finding it very difficult on present income	12 (2.5)
Finding it difficult on present income	37 (7.9)
Getting by on present income	125 (26.5)
Living comfortably on present income	284 (60.3)
Prefer not to say	13 (2.8)
RUCA Code^a^	
Metropolitan	228 (48.4)
Micropolitan	127 (27.0)
Rural	111 (23.6)
No data	5 (1.1)
MVPA^b^, minutes per week, median (IRQ)	150 (80–280)
Combustible cigarette use	
Never smoker	277 (58.8)
Former smoker	182 (38.6)
Current smoker	12 (2.5)
Alcohol use	
Never consumer	24 (5.1%)
Former consumer	137 (29.1%)
Current consumer	310 (65.8%)
Type of primary cancer, *n* (%)	
Breast	129 (27.4%)
Prostate	94 (20.0%)
Melanoma skin	62 (13.2%)
Non-Hodgkin's lymphoma	18 (3.8%)
Cervical	17 (3.6%)
Thyroid	17 (3.6%)
Colorectal	14 (3.0%)
Other^c^	137 (29.0%)
Alcohol-related primary cancer, *n* (%)	161 (34.2%)
Age at first diagnosis in years, mean ± SD	56.5 ± 13.2
Time since first diagnosis in years, mean ± SD	12.9 ± 11.2
Years since the first cancer diagnosis, *n* (%)^d^	
≤ 5 years	150 (32.5%)
>5, ≤ 10 years	90 (19.5%)
>10 years	221 (47.9%)
Currently in treatment, *n* (%)	65 (13.8%)
Disease progression, *n* (%)	38 (8.2%)
No evidence of disease, *n* (%)	106 (22.5%)
FACT-G7, mean (SD)	18.6 ± 2.9

^a^RUCA, rural-urban commuting area; range from one (metropolitan) to 10 (remote rural). RUCA codes 1–3 defined as metro, codes 4–6 defined as micro, and 7–10 defined as rural.

^b^MVPA, moderate to vigorous physical activity.

^c^Other includes head, neck, and/or throat cancer (*n* = 14, 3.0%), bladder cancer (*n* = 13, 2.8%), lung cancer (*n* = 10, 2.1%), basal cell carcinoma (*n* = 8, 1.7%), chronic lymphocytic leukemia (*n* = 8, 1.7%), kidney cancer (*n* = 8, 1.7%), ovarian cancer (*n* = 8, 1.7%), endometrial cancer (*n* = 6, 1.3%), leukemia (*n* = 6, 1.3%), testicular cancer (*n* = 4, 0.8%), esophageal cancer (*n* = 3, 0.6%), appendix cancer (*n* = 2, 0.4%), Hodgkin's lymphoma (*n* = 2, 0.4%), pancreatic cancer (*n* = 2, 0.4%), squamous cell carcinoma (*n* = 2, 0.4%), squamous cell carcinoma of the eyelid (*n* = 2, 0.4%), anal cancer (*n* = 1, 0.2%), brain cancer (*n* = 1, 0.2%), chondrosarcoma (*n* = 1, 0.2%), chronic myeloid leukemia (*n* = 1, 0.2%), dermatofibrosarcoma (*n* = 1, 0.2%), epithelioid hemangioendothelioma (*n* = 1, 0.2%), gastrointestinal stromal tumor (*n* = 1, 0.2%), leiomyosarcoma (*n* = 1, 0.2%), liposarcoma (*n* = 1, 0.2%), liver cancer (*n* = 1, 0.2%), Merkel cell carcinoma (*n* = 1, 0.2%), mesothelioma (*n* = 1, 0.2%), mucoepidermoid carcinoma (*n* = 1, 0.2%), multiple myeloma (*n* = 1, 0.2%), myelodysplastic syndrome (*n* = 1, 0.2%), nasal mucosal melanoma (*n* = 1, 0.2%), not stated (*n* = 1, 0.2%), ocular melanoma (*n* = 1, 0.2%), sarcoma (*n* = 1, 0.2%), soft tissue sarcoma (*n* = 1, 0.2%), stomach cancer (*n* = 1, 0.2%), and Waldenström's macroglobulinemia (*n* = 1, 0.2%).

^d^Data missing for 10 participants, totals are based on 461 records.

The most frequently reported primary cancer types were breast (27.4%), prostate (20.0%), and melanoma of the skin (13.2%), followed by a heterogeneous group of less common malignancies. Approximately one-third of the sample (34.2%) were diagnosed with an alcohol-related primary cancer. The mean age at first cancer diagnosis was 56.5 years (SD = 13.2), with an average of 12.9 years since diagnosis (SD = 11.2).

### Current and past alcohol consumption patterns

The majority of participants (65.8%) reported currently consuming alcohol, while 29.1% were former consumers and 5.1% had never consumed alcohol in their lifetime (Table 1). When comparing sociodemographic characteristics between current alcohol consumers and non-consumers (former and never alcohol users combined), we observed significant differences across multiple factors. Current alcohol consumers were more likely to have obtained postgraduate education compared to non-consumers (51.1 vs. 41.0%; *p* = 0.02). Additionally, a higher proportion of current consumers reported living comfortably on their present income (67.7 vs. 51.0%; *p* < 0.001). Conversely, current alcohol consumers were slightly less likely to be current smokers compared to non-consumers (1.3 vs. 5.0%; *p* = 0.048). They also engaged in more moderate-to-vigorous physical activity per week, with a median of 180 min compared to 135 min among non-consumers (*p* = 0.06).

In addition to sociodemographic variation, comparisons of cancer history characteristics revealed differences between current alcohol consumers and non-consumers; current consumers reported a slightly lower average quality-of-life score, as measured by the FACT-G7 (18.4 vs. 19.0; *p* < 0.039). No other differences in cancer history characteristics between the two groups reached the *p* < 0.05 threshold, including having an alcohol-related primary cancer.

Among current alcohol consumers, the median number of drinks per week was four (IQR: 2–8); 13.5% exceed the moderate drinking threshold. Former alcohol consumers reported a median of three drinks per week (IQR: 1.25–8.0) during their period of active consumption; 20.4% exceeded the moderate drinking threshold before cessation.

### Awareness of alcohol-related cancer risk and efforts to reduce consumption

Awareness that alcohol use increases the risk of developing cancer was modest: 49.7% of current alcohol users and 48.2% of former alcohol users responded they were aware of alcohol as a cancer risk factor, while approximately one-third of each group responded “no,” and the remainder expressed uncertainty (Table 2). When asked whether a healthcare provider had discussed strategies to reduce or quit alcohol consumption, only 7.7% of current consumers reported receiving such guidance, compared to 19.7% of former consumers (*p* < 0.001). Furthermore, 10.0% of current consumers reported reducing their alcohol consumption because it is a cancer risk factor, while 26.1% of former consumers had done so (Table 2).

**Table 2:** Awareness of alcohol as a cancer risk factor and efforts to reduce alcohol drinking by former and current consumers (*n* = 447)^a^.

Survey item/Question	Among former alcohol consumers (*n* = 137)	Among current alcohol consumers (*n* = 310)	*p*-value
Before today, did you know alcohol is a cancer risk factor?			
Yes	66 (48.2%)	154 (49.7%)	0.749
Maybe	22 (16.1%)	55 (17.7%)	
No	49 (37.8%)	101 (32.6%)	
Affirmative response that alcohol increases cancer risk, by type:			
Liquor/spirits	74 (54.0%)	153 (49.5%)	0.439
Beer	61 (44.5%)	115 (37.2%)	0.176
Wine	51 (37.5%)	111 (35.9%)	0.832
A health care provider has discussed ways to reduce or quit drinking alcohol, either in general or as part of cancer care	27 (19.7%)	24 (7.7%)	< 0.001
Reduced or stopped drinking alcohol after diagnosis because alcohol is related to developing certain cancers^b^			
Yes	35 (26.1%)	31 (10.0%)	< 0.001
A little bit	8 (6.0%)	46 (14.9%)	
No	91 (67.9%)	232 (75.1%)	
Any past efforts to reduce alcohol intake, not just efforts related to cancer risk, *n* (%)	85 (64.4%)	132 (42.7%)	< 0.001
Would like to reduce alcohol consumption in the next 6 months, *n* (%)^c^			
No	–	195 (63.1%)	–
Not sure	–	32 (10.4%)	
Maybe yes	–	59 (19.1%)	
Definitely yes	–	23 (7.4%)	

^a^Data presented includes only former and current alcohol consumers; individuals who have never consumed alcohol are not represented.

^b^This question was only asked to participants who answered ‘*Definitely yes'* or ‘*Maybe yes'* to knowing alcohol is a cancer risk factor. Participants who responded ‘*No*' were classified as having made no past efforts to reduce or stop drinking due to cancer risk.

^c^Data was missing for one current alcohol user in this category.

### Intention to reduce alcohol use and associated factors

Among current alcohol consumers (*n* = 310), 26.5% reported they would like to reduce their intake within the next 6 months (including 19.1% who expressed that they might reduce their use and 7.4% who definitely planned to do so). Fewer (10.4%) were unsure (Table 2). Comparative analyses between individuals with and without intentions to reduce alcohol found that those with intentions to reduce were younger on average (mean age 67.6 ± 11.2 vs. 70.8 ± 9.3 years; *p* = 0.022, consumed significantly more drinks per week (median 7.0 vs. 3.0 drinks; *p* < 0.001), and were more likely to exceed moderate-drinking thresholds (25.0 vs. 9.8%; *p* = 0.001). They also had higher median AUDIT scores (4.0 vs. 3.0; *p* < 0.001) and a greater prevalence of AUDIT ≥ 8 (23.2 vs. 4.0%; *p* < 0.001), reflecting more hazardous drinking patterns (Table 3). No significant differences were observed in sex distribution between the groups (*p* > 0.99).

**Table 3:** Alcohol use characteristics among cancer survivors in New Hampshire and Vermont who consume alcohol, stratified by intentions to reduce or stop use.

Alcohol use characteristics	Overall	Stratified by intentions to reduce alcohol use^e^		
	Current alcohol consumers (*n* = 310)	Current alcohol consumers with intentions to reduce use^a^ (*n* = 82)	Current alcohol consumers without intentions to reduce use (*n* = 227)^e^	*p*-value
Drinks per week^b^, median (IQR)	4.0 (2.0, 8.0)	7.0 (3.8, 7.8)	3.0 (1.0, 7.0)	< 0.001
Exceeding moderate drinking per week^c^, *n* (%)	42 (13.5%)	20 (25.0%)	22 (9.8%)	0.001
>2 drinks on days when drinking, *n* (%)	36 (11.6%)	15 (18.3%)	21 (9.3%)	0.047
Binge drinking, *n* (%)	39 (12.6%)	15 (18.3%)	24 (10.6%)	0.111
FACT G7, mean ± SD	18.4 ± 2.8	18.5 ± 2.7	18.1 ± 3.1	0.351
AUDIT^d^ score, median (IQR)	4.0 (2.0, 4.0)	4.0 (3.3, 6.8)	3.0 (2.0, 4.0)	< 0.001
AUDIT^d^ score ≥ 8, *n* (%)	28 (9.0%)	19 (23.2%)	9 (4.0%)	< 0.001
AUDIT^d^ score ≥ 15, *n* (%)	1 (0.3%)	1 (1.2%)	0 (0.0%)	–

^a^Intentions to reduce or stop alcohol use were defined as responding ‘*Yes*' or ‘*Maybe yes*' when asked whether they would like to reduce their alcohol consumption in the next 6 months (compared to ‘*No*' or ‘*Not sure*').

^b^IQR: Interquartile range. Data on drinks per week was missing for six current alcohol users. These cases were excluded from the analysis.

^c^Exceeding moderate drinking was defined as consuming more than 14 drinks per week for men and more than 7 drinks per week for women. Data on drinks per week was missing for six current alcohol users.

^d^AUDIT score data was missing for one current drinker and was excluded from the analysis.

^e^Data was missing for one current alcohol user in this category.

### Perceived barriers to reducing alcohol consumption

**Figure 1:**
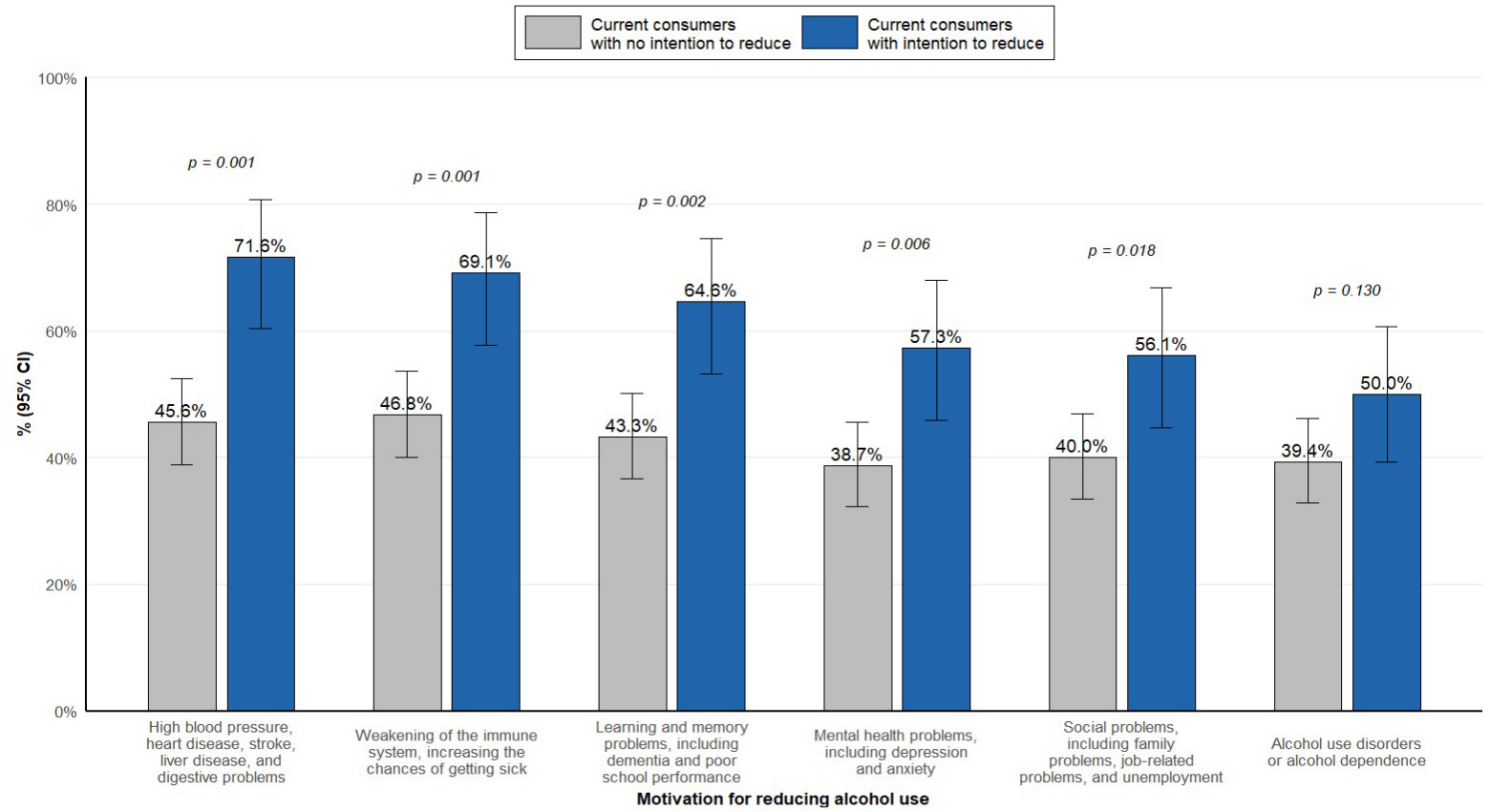
Proportion of current alcohol users endorsing each of six reasons to reduce alcohol use as important to very important Response options were dichotomized as “important/very important” vs. all other categories. Reported *p*-values correspond to between-group comparisons for each reason.

Among current alcohol users who intended to reduce their drinking, health-related risks were the most frequently endorsed reasons to cut down: high blood pressure/cardiovascular disease and other medical problems (71.6%), weakening of the immune system (69.1%), and learning and memory problems (64.8%). Each of these reasons was cited significantly more often than among users without intentions to reduce (*p* = 0.001, *p* = 0.001, and *p* = 0.002, respectively; Figure 1). Mental health problems (57.3%; *p* = 0.006) and social problems (56.1%; *p* = 0.018) were also more commonly endorsed by those intending to reduce. In contrast, alcohol use disorder/dependence was less frequently cited (50%) and did not differ significantly by reduction intention (*p* = 0.130).

**Figure 2:**
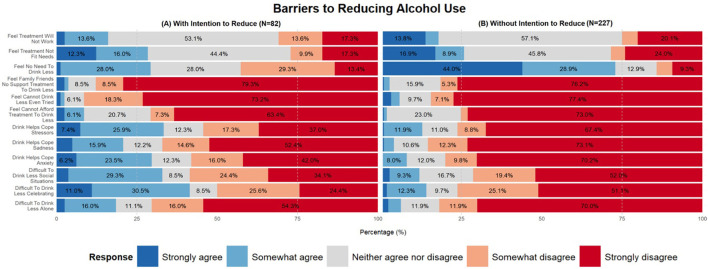
Perceived barriers to reducing alcohol consumption among cancer survivors in New Hampshire and Vermont who consume alcohol. Stacked bars display the full 5-point Likert distribution (strongly agree to strongly disagree). In the Results text, percentages correspond to collapsed agreement (somewhat agree + strongly agree) computed from the analytic dataset; very small segments may be unlabeled in the graphic due to space/rounding. **Left panel**: with intention to reduce (*n* = 82). **Right panel**: without intention to reduce (*n* = 227); Data was missing for one current alcohol user in this category.

Survivors who reported they would like to reduce their alcohol use (Figure 2) endorsed psychosocial factors as barriers: 41.5% agreed that “drinking while celebrating” would impede reduction (vs. 14.1% of non-intenders) and 32.9% reported difficulty “drinking less in social settings” (11.9% of non-intenders). Emotional-coping motives were prevalent: 33.3% of intenders agreed that “drinking helps cope with stressors” and 29.7% cited using alcohol to cope with anxiety or 20.7% sadness. Endorsements of those items as barriers were considerably lower among those without intentions to reduce their drinking. Most strikingly, 72.9% of non-intenders “feel no need to drink less,” compared with 29.3% of intenders, and non-intenders less frequently endorsed social or emotional barriers (all < 20%). Fewer than 5% of all current alcohol users endorsed cost or a lack of social-support as a barrier to reducing alcohol use. Percentages reflect collapsed agreement (somewhat + strongly agree) computed from the analytic dataset.

### Comparisons by alcohol-related primary cancer status

Exploratory analyses stratified by whether the primary cancer was alcohol-related indicated greater risk salience among survivors with an alcohol-related cancer: 51.4% reported reducing or stopping drinking because of cancer risk compared with 35.5% of survivors with other cancers (*p* = 0.010), and awareness that alcohol increases cancer risk was higher, though not statically significantly so, among survivors with an alcohol-related cancer (54.7 vs. 45.2%; *p* = 0.063). Provider counseling about alcohol reduction did not differ by cancer type (11.4 vs. 12.2%; *p* = 0.954). Current alcohol use did not differ significantly by alcohol-related cancer status (64.0 vs. 66.8%; *p* = 0.614) and any past effort to reduce alcohol intake was similar between groups (50.3 vs. 47.7%; *p* = 0.667). Among current consumers (*n* = 103 alcohol-related; *n* = 207 other), intention to reduce drinking within the next 6 months was comparable (26.2 vs. 26.7%; *p* = 1.00). Among current consumers, self-reported alcohol dependence (AUDIT ≥ 15) was rare and did not differ significantly by cancer type (1.9 vs. 0.0%; *p* = 0.208). Quality of life was also similar (FACT-G7 mean, 18.5 vs. 18.7; *p* = 0.447; median (IQR), 19.0 (17.0–20.0) in both groups; *p* = 0.197). Percentages reflect valid non-missing responses; these stratified comparisons were prespecified as exploratory and *p* values were not adjusted for multiple testing.

## Discussion

This study provides a robust characterization of self-reported alcohol consumption patterns, awareness of oncologic risks, and behavioral intentions regarding alcohol reduction among a sample of cancer survivors in New Hampshire and Vermont who reside in the catchment area of our Cancer Center. Despite national and international recommendations encouraging alcohol reduction among individuals with a history of cancer (19, 33, 42, 43), two third of survivors in our sample reported current alcohol use. Although overall drinking patterns were largely moderate, among current alcohol consumers, 13.5% exceeded sex-specific moderate-drinking threshold and 9.0% had AUDIT scores ≥8, indicating hazardous drinking. These findings are notable given that 34.2% of participants had a history of alcohol-related cancer specifically. National Claims-based studies provided related context: among commercially insured U.S. cancer survivors, diagnosed AUD prevalence increased from 0.78% in 2012 to 1.43% in 2021, and only 14.3% of survivors with an AUD diagnosis started treatment within 1 year (44, 45). In stratified comparisons by alcohol-related primary cancer status, current use and six-month intentions were similar between groups, whereas survivors with an alcohol-related cancer more often reported reducing or stopping because of cancer risk. As this sample largely reflects a long-term survivor population, these findings support the importance of addressing alcohol use throughout survivorship as part of healthy aging.

This study found that, even at moderate levels of drinking, many survivors indicated a desire to reduce their alcohol use within the next 6 months. In our sample, 23.2% of alcohol users intending to cut back met the AUDIT threshold for hazardous drinking (≥8), compared with just 4.0% of those without such intentions. This aligns with findings from Alalwan et al. (1), who reported similarly “moderate” reduction intentions among breast cancer survivors aware of the alcohol–cancer link (46). In comparison, Seth et al. (48), observed that only 10% of survivors actually ceased alcohol after diagnosis while 45% maintained and 34% increased their intake (47). Previous studies have demonstrated that hazardous alcohol users often face clinical and psychological obstacles that impede their ability to reduce intake, such as symptoms of alcohol dependence and the use of alcohol as a coping strategy (16, 48). However, dependence rates (AUDIT ≥15) in our sample were very low, indicating that most individuals meeting AUDIT ≥8 represented moderate hazardous alcohol users rather than clinically dependent users. These findings emphasize the need for interventions to address the multiple health benefits of reducing alcohol use among moderate alcohol users who likely do not identify as someone at risk of alcohol dependence.

A notable finding in our study was that only 7.7% of current alcohol users reported having discussed alcohol reduction with a healthcare provider, compared to 19.7% of former alcohol users. In fact, Greene et al. (19) found that, although 78.4% of cancer survivors recalled being screened for alcohol use, only 58.7% had an in-person discussion about cutting back and just 15% of heavier alcohol users were advised to reduce their intake (49). More recent national evidence further reinforces this gap: Check et al. (13) found that only approximately 40% of cancer survivors received any alcohol screening and that, among those with unhealthy alcohol use, survivors were no more likely than adults without cancer to receive brief intervention (~8%) or treatment referral (~2%) (50). In our sample, most former (67.9%) and current (75.1%) alcohol users similarly reported that they had not reduced their alcohol use because it was a cancer risk factor. International guidelines, including recommendations from the U.S. Preventive Services Task Force (USPSTF), advocate universal alcohol screening and brief interventions in clinical settings (51). However, cancer-specific guidance remains limited. The National Comprehensive Cancer Network (NCCN) Survivorship Guidelines advise survivors to avoid or strictly limit alcohol consumption, especially among individuals treated for cancer of the head and neck, liver, and esophagus (11). Similarly, the American Society of Clinical Oncology (ASCO) recognizes alcohol as a modifiable behavioral risk factor and encourages oncologists to raise awareness and engage in conversation about alcohol use during and after cancer treatment (52). The American Cancer Society also advises that it is best to avoid alcohol due to its association with increased cancer risk (53). These findings underscore a critical gap between recommendations and clinical practice and call for more intentional, tailored discussions around alcohol use in survivorship care.

This disconnect is also reflected in broader public health patterns. Nationally, only 44% of U.S. adults recall discussing alcohol-related harms with a clinician (49). When such conversations do occur, they often emphasize quantity but neglect frequency, consequences, or treatment strategies. Consistent with these observations, in our sample, only 10% of current alcohol users reported reducing their alcohol intake because of cancer risk, 14.9% reported a slight reduction, and 75.1% reported no change (Table 2). A comparable gap was reported by Greene et al. (19), in which fewer than 10% of excessive current alcohol users receive advice to reduce their drinking, reinforcing the urgent need for more consistent and impactful alcohol counseling across healthcare settings.

Findings from this survey are useful to understand alcohol use behaviors among survivors within our Cancer Center's catchment area, and findings support that providers may be an underutilized resource for increasing the awareness of alcohol as a cancer risk factor among those with cancer. A Canadian study found that survivors who received formal counseling were six times more likely to reduce their consumption (54). A cancer diagnosis may also offer a “teachable moment”, when self-reflection on personal values and priorities can motivate healthier lifestyle behaviors (55–57). However, longitudinal evidence suggests that this effect may be limited for alcohol use. Kiefer et al. (22) found that approximately 70% of patients with cancer continued drinking after diagnosis, with risky drinking remaining common over 6 months of psycho-oncological care, and higher anxiety levels associated with risky consumption (58). Cancer survivors also have frequent contact with the health care system, creating opportunities for intervention. In a nationally representative survey during 2015–2019, 58.7% of adult cancer survivors with an in-person interaction with a healthcare provider in the prior 12 months reported that a professional initiated a discussion about alcohol use (49).

The current study did not find a difference in current alcohol use between survivors with an alcohol-related primary cancer and those with other cancers (64.0 vs. 66.8%; *p* = 0.614). Prior work has shown high-risk drinking among survivors of alcohol-related cancer −100%in esophageal, 71.4% in rectal, 62.5% in laryngeal, 38.6% in colon, and 35.7% in liver—compared with 24.9% among survivors of cancer not associated with alcohol (48). Only one third of our cohort had been diagnosed with an alcohol related cancer, likely reflecting a sample of healthier, long-term survivors; 67.4% were more than 5 years from their initial diagnosis. In our data, indicators of problematic drinking were generally low, and overall use reflected moderate patterns. Current consumers reported higher socioeconomic status and greater physical activity, although FACT-G7 score were slightly lower among current consumers than non-consumers (18.4 vs. 19.0%; *p* = 0.039). This pattern is partially consistent with prior survivor cohorts showing that current alcohol use clusters with higher socioeconomic status, greater activity, and better quality of life (19, 20, 52). Longitudinal evidence further demonstrates that financial stability and greater physical activity predict sustained alcohol consumption after a breast cancer diagnosis (30), reinforcing that interventions should not overlook survivors who appear healthy and financially secure, and is typically attributed to selection (healthy user and sick-quitter effects) rather than any protective effect of alcohol, which aligns with the near absence of AUDIT ≥15 in our cohort. Even so, a considerable proportion of participants expressed a desire to reduce their alcohol intake within the next 6 months. The lower prevalence of current smoking among current alcohol consumers (1.3 vs. 5.0%; *p* = 0.048) in this sample may reflect the sociodemographic profile of this subgroup. In U.S. adults, current smoking is less common among those with higher educational attainment and socioeconomic advantage (59).

In addition to motivations for behavior change, behavioral interventions must also address barriers to change. Participants in this study endorsed various barriers, including social reasons such as alcohol use during celebrations and emotional reasons such as managing stress, anxiety, or sadness. These barriers reflect both behavioral and psychological dimensions, highlighting the need for broader psychoeducational frameworks in survivorship care. Structural barriers such as cost, access, or perceived ineffectiveness of treatment were less frequently cited. Notably, the most common barrier among those with no intention to reduce was the belief that they “do not need to drink less,” pointing to a low perception of risk. These findings support the need for multilevel interventions that combine targeted education about alcohol's health effects with efforts to shift social norms around alcohol use.

Our results also align with literature suggesting that moderate alcohol users represent a large portion of the population who may benefit more from broad public health interventions (60). While heavy alcohol users often require specialized treatment, moderate alcohol users contribute substantially to the cancer burden attributable to alcohol. It is estimated that moderate drinking accounts for one in every seven alcohol-related cancer cases (6), implying that strategies aimed at reducing average consumption could be more impactful than focusing solely on extreme use. In our study, cardiovascular and memory harms were the most commonly endorsed reasons for cutting back (~70%), whereas concern about alcohol dependence was supported by about half. Emphasizing alcohol's overall health impacts may therefore be a practical motivator for reduction among otherwise healthy, long-term cancer survivors.

This study leveraged a population-based sample of cancer survivors from two states with similar sociodemographic and healthcare system profiles, enhancing the external validity of our findings at the regional level. We used validated instruments (AUDIT, FACT-G7) and assessed alcohol use patterns (current use, frequency, quantity, AUDIT scores and hazardous-drinking thresholds) alongside motivational constructs (intention to reduce drinking within 6 months, perceived health risks and reasons for reduction, and recall of provider counseling). However, the study presents several limitations. Its cross-sectional design limits causal inferences or assessment of pre- vs. post-diagnosis changes in alcohol use. Self-reported variables may be influenced by social desirability bias, especially regarding alcohol consumption and risk perception. Prior studies have shown that individuals seeking to present a favorable image tend to underreport their actual consumption by 20–33% and underestimate consequences by approximately 50%. Our sample's racial/ethnic composition was overwhelmingly White (99.8%), which limits generalizability to more diverse populations and precluded meaningful subgroup analyses by race/ethnicity. The sample also included a high proportion of socioeconomically advantaged survivors and metropolitan residents, which may limit generalizability to underserved populations. The opt-in, online panel design is also likely to underrepresent survivors with more severe illness, limited digital access, or lower socioeconomic status.

Alcohol consumption was prevalent among our population of cancer survivors. While most of those who did consume alcohol did so at moderate levels, a considerable portion of those survivors expressed the desire to reduce or quite alcohol use over the next 6 months. General health concerns were rated as top motivations for reducing alcohol use, suggesting that interventions could be tailored to this population of long-term and relatively health survivors with such themes. Overall, participants recall of discussing their alcohol use with a cancer provider was low, highlighting a potential intervention point where survivor awareness of alcohol's impact on health and wellbeing could be addressed.

## Data Availability

The raw data supporting the conclusions of this article will be made available by the authors, upon reasonable request; sharing the data requires an IRB-approved research plan.
